# Giant multinucleated plasma cells in relapsed plasma cell myeloma

**DOI:** 10.1002/jha2.35

**Published:** 2020-07-13

**Authors:** Sylvia Ghattas, Jonathan Salisbury

**Affiliations:** ^1^ Department of Histopathology King's College Hospital Denmark Hill London


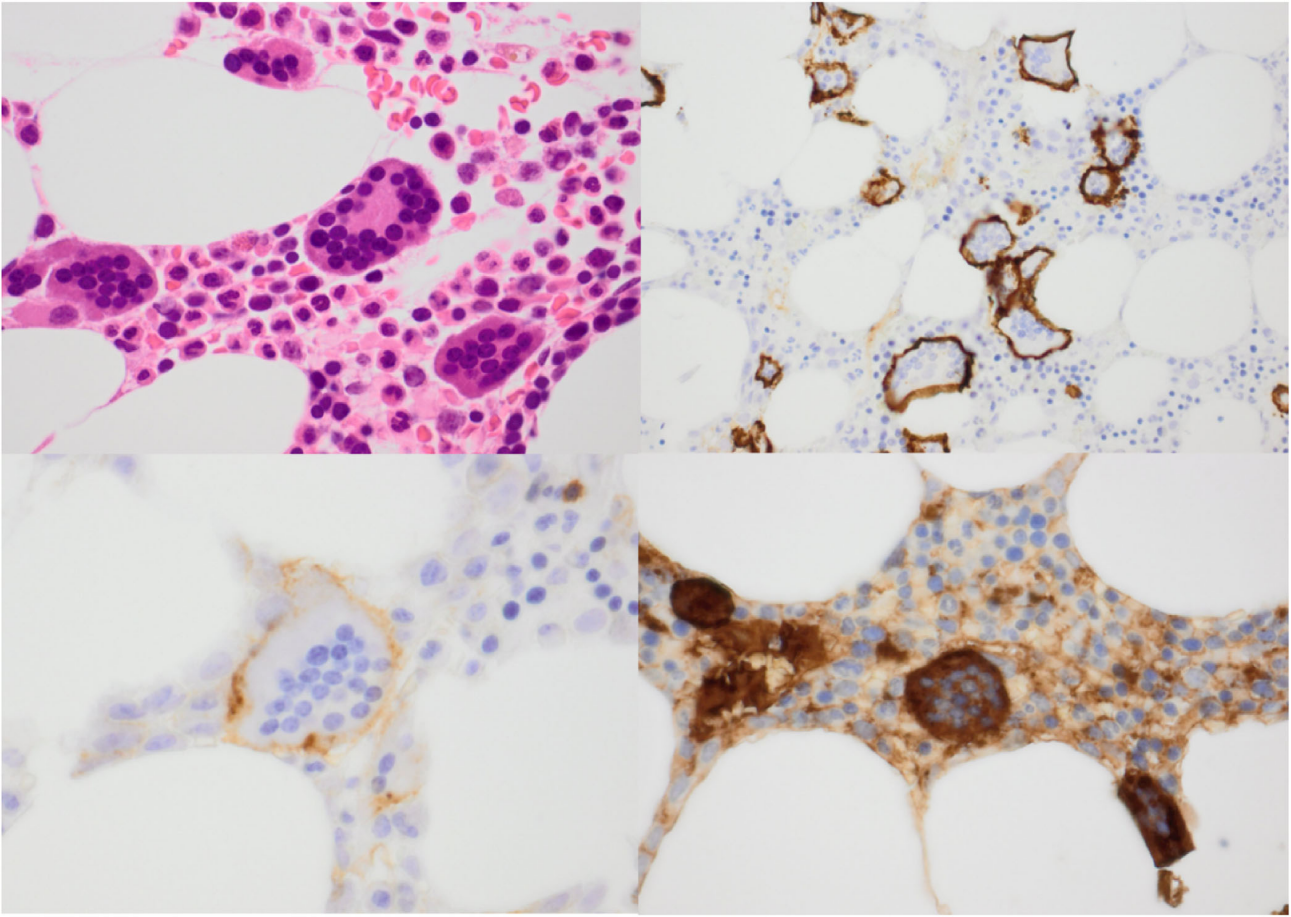


A 47‐year‐old woman was diagnosed with plasma cell myeloma in 2018, received first‐line induction chemotherapy, and made a partial response. She then received two cycles of DT‐PACE chemotherapy. Her paraprotein levels remained static and a repeat bone marrow test in February 2019 revealed persistent bone marrow involvement by plasma cell myeloma showing usual (mononuclear) morphology with no multinucleated giant cells. She was treated with daratumumab as a bridge to an autologous stem cell transplant in June 2019. Her day 100 post autograft bone marrow trephine biopsy contained a plasma cell population making up 10% of all nucleated cells. More than 95% of the atypical plasma cells showed multinucleated giant cell morphology containing up to 40 nuclei. On immunohistochemistry, these multinucleated giant cells showed positive staining for CD138, CD38, and CD56 and were lambda restricted. CD45 was negative.

Bone marrow trephine sections stained (clockwise from top left) with H&E, and for CD138, lambda light chains, and CD38.

Multinucleated tumor giant cells are thought to form by two mechanisms: by cell‐cell fusion via syncytins or as a result of a failure of cytokinesis at the end of the anaphase stage of mitosis. Occasional case reports of giant multinucleated plasma cells have been published since the 1970s. However, the presence of giant plasma cells with more than 10 nuclei is extremely rare. One previous case had also been treated with daratumumab, an IgG1k monoclonal antibody directed against cell surface CD38 (Guijarro F, Rozman M, Matutes E. Multinucleated giant myeloma cells after failure of daratumumab therapy. *Br J Haematol* 2018;181:432). Plasma cell membrane alterations promoting cell‐cell fusion may be a side effect of immunotherapies targeting cell surface molecules. Giant multinucleated plasma cells may be observed more frequently as such immunotherapies become increasingly used.

